# Resolution of otitis media in a humanized mouse model

**DOI:** 10.3389/fgene.2022.958540

**Published:** 2022-11-09

**Authors:** Ye Lin Son, Kwang Pak, Nada Muradagha, Kyung Wook Heo, Anke Leichtle, Arwa Kurabi

**Affiliations:** ^1^ Department of Surgery/Otolaryngology, School of Medicine, University of California San Diego, La Jolla, CA, United States; ^2^ Department of Otorhinolaryngology, Head and Neck Surgery, Inje University Busan Paik Hospital, Busan, Korea; ^3^ Department of Otorhinolaryngology, Head and Neck Surgery, University Hospital Schleswig-Holstein, Lübeck, Germany

**Keywords:** middle ear, leukocytes, inflammation, humanized mice, animal models otitis media, transgenic mice chimera

## Abstract

Otitis media (OM) is one of the largest public health problems of children and has devastating impacts in developing countries. The substantial medical and human costs involved have led to research to understand the disease and improve treatment. Animal models of OM have yielded critical information about the immune, inflammatory and genetic mechanisms of OM. However, it is important to link animal studies to human immune and inflammatory responses. In recent years, “humanized” mice have become a valuable tool to study the human immune system in an animal model. Here we describe the first use of humanized mice to study OM. We demonstrate that humanized mice with a sufficient degree of engraftment recapitulate a normal middle ear (ME) inflammatory response to bacterial infection, including the recruitment of human immune cells, and exhibit normal recovery. Moreover, these animals exhibit regulated expression of human-specific immune and inflammatory genes in the ME. In contrast, mice with insufficient engraftment fail to resolve OM. This model has many potential uses in OM research, including using hematopoietic stem cells from patients with differing degrees of OM susceptibility, to understand the role of human immune responses in proneness to this common childhood disease.

## Introduction

Otitis media (OM), infection and inflammation of the middle ear (ME), is a highly prevalent childhood disease with significant morbidity in developed and especially developing countries (Klein, 2000; Acuin, 2004). While over 85% of children experience OM, ∼10–15% suffer from recurrent or persistent ME disease (Casselbrant and Mandel, 2001; Rovers, 2008; Rye et al., 2012; Thomas and Brook, 2014; Kaur et al., 2016) and are termed otitis-prone. Early hearing loss from OM has been linked to speech/language delays (Friel-Patti and Finitzo, 1993), learning deficits (Teele et al., 1989; Williams and Jacobs, 2009) and disorders of auditory processing (Graydon et al., 2017). In the developed world, OM consumes considerable health care resources. In the United States, the estimated costs trail $5 billion (Tong et al., 2018). In developing countries, undertreated OM accounts for an estimated 28,000 annual deaths and half of the world’s burden (∼175 million cases) of serious hearing loss (Acuin, 2004; World Health Organizaiton, 2021).

OM is associated with infection of the ME by bacteria including non-typeable *Haemophilus influenzae* (NTHi), *S. pneumoniae*, and *Moraxella catarrhalis*. OM pathology is characterized by inflammation leading to ME mucosal hyperplasia and recruitment of leukocytes into the ME (Lim and Birck, 1971). Although OM is multifactorial, with contributions from Eustachian tube dysfunction, general health and environmental factors, recent evidence implicates innate and immune responses in acute OM pathogenesis and resolution (Mittal et al., 2014), while adaptive immunity is important for long-term resistance (Schilder et al., 2016; Kyd et al., 2017).

Current animal models of OM have provided significant insights into potential molecular mechanisms of pathology and resolution. This includes studies in the mouse (Trune and Zheng, 2009; Tyrer et al., 2013), chinchilla (Bakaletz, 2009), guinea pig (Sato, 1997), rat (Hermansson et al., 1988), and monkey (Dohar et al., 2005). Animal studies take advantage of the ability to monitor OM through all of its stages, obtain tissue samples at will, and use genetically modified animals to explore the role of specific genes and gene variants, including gene deletions. They have increased understanding of the contributions of the Eustachian tube (Lim et al., 2000; Piltcher et al., 2002), innate immunity (Leichtle et al., 2011) and host genetics (Rye et al., 2011; MacArthur et al., 2013) to OM pathogenesis and recovery (Hernandez et al., 2015). In addition, significant progress in adaptive immune responses to OM pathogens and OM vaccines has been achieved through animal studies (Cripps and Kyd, 2003; Sabirov and Metzger, 2008).

While animal studies illuminate the roles of different immune and non-immune processes throughout the course of OM, data on human pathways is generally restricted to active disease. A common critique of animal models is the reliance on non-human immune responses to infection. For example, laboratory mouse strains can differ significantly in their susceptibility to the same bacterial pathogen, including in the ME (Melhus and Ryan, 2003). Accordingly, while human pathogens such as *S. pneumoniae* and NTHi induce OM in laboratory animals, these animals are not readily colonized by others like *Moraxella catarrhalis* (Melhus and Ryan, 2003; Goldstein et al., 2009). Thus the interpretation of results from animal studies can be limited by the degree to which animal immunity differs from that of humans. These differences include immune molecules that are lacking in or unique to mice, species differences in immune and host defense processes, and interactions with human pathogens that have evolved under human immune pressure (Rehli, 2002; Warren et al., 2010; Shay et al., 2013). There is currently debate about the degree to which human and mouse models of inflammation can be compared (Seok et al., 2013; Takao and Miyakawa, 2015). However, the ability to study human immune cells in an animal setting would allow us to address such issues more directly, while preserving the many advantages of animal models. A recent approach to this issue are immune-compromised mouse models into which human immune cells have been engrafted to produce “humanized” mice (Shultz et al., 2012).

Recent humanized mouse advances include the use of severely immunodeficient NOD-SCID-IL2Rγ (NSG) mice, which have very limited ability to mount immune responses. This allows superior reconstitution of the immune system with human hematopoietic stem cells (HSCs), giving rise to mice with a human hematolymphoid immune system (humanized-NSG mice: huNSG). Over time, the engrafted HSC undergo multilineage development, resulting in a fully functional human immune system, including T-, B-, NK- and dendritic cells, as well as monocytes/macrophages and granulocytes (Pearson et al., 2008; Shultz et al., 2010). Moreover, residual murine immune cells are virtually non-functional, so observed immune responses are limited to those produced by human cells. Engrafted human immune cells have been shown to mediate human-specific immune responses in many diseases, including arthritis, diabetes, cancer, HIV, and sepsis, as well as in immunologic drug testing [reviewed in (Walsh et al., 2017; Mian et al., 2021)].

The aims of the present study were to explore the potential of humanized mouse models for the study of OM, to determine whether these mice respond similarly to established mouse OM models, to compare their responses to the parental BALB/c strain, and to document human immune responses to infection in the mouse ME. The NSG background carries several genetic defects primarily affecting innate and adaptive immune responses. NSG mice lack immune responses to bacterial and fungal pathogens and have been reported to spontaneously develop OM (Santagostino et al., 2017). We performed a series of experiments to demonstrate that humanized NSG mice recapitulate the normal ME inflammatory response to infection and that the mobilization of human immune cells into the circulation and trafficking into the ME is responsible for OM resolution and bacterial clearance. Finally, we demonstrate the expression of human-specific immune genes in the humanized mouse ME during OM.

## Materials and methods

### Animals

**FIGURE 1:**
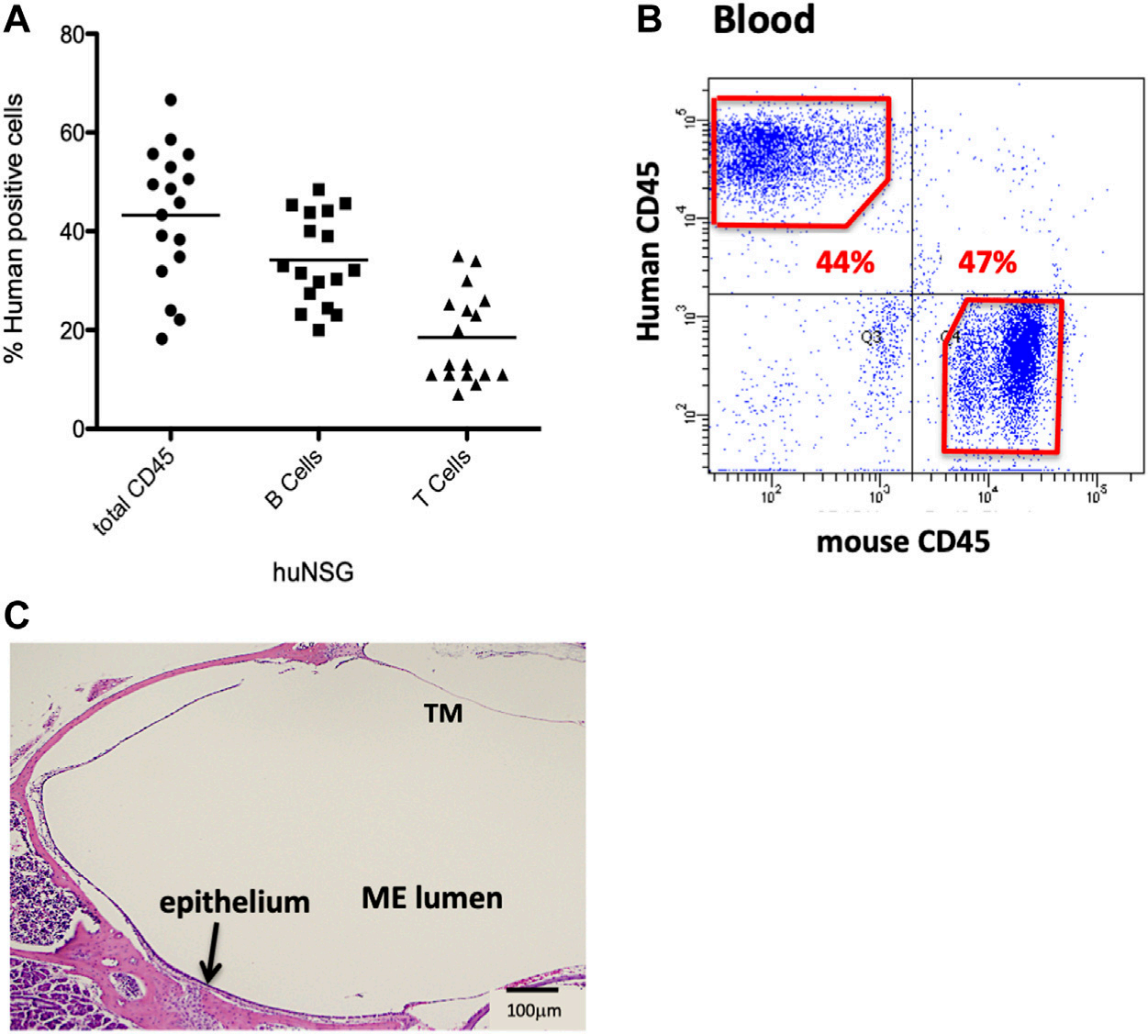
Characterization of the NSG mice reconstituted with human CD34^+^ HSPCs. **(A)** Average % human CD45^+^ cells present 12 weeks post-engraftment. **(B)** Sample flow cytometry analysis of hCD45^+^ vs. mCD45^+^ cells present in blood of huNSG mice. **(C)** Histopathology of the middle ear (ME) and inner ear of huNSG mice prior to any experimental challenge showing the mucosa of the normal ME.

Humanize mice (huNSG) were purchased from Jackson Labs (Bar Harbor, ME). In brief, these immunodeficient 2–3 weeks old female NOD-SCID-IL2Rγ^−/−^ (NSG) mice had been irradiated for myelo-ablation at a sub-lethal dose, and then reconstituted with CD34^+^-HSCs from human fetal liver, by intravenously injection *via* the tail vein. Degree of engraftment had been evaluated by flow cytometry using antibodies against human CD45, T-cells and B-cells, 12 weeks post-injection, and reported to be >20% circulating human leukocytes expressing the human CD45 (hCD45^+^) antigen (Figure 1A).

Animals were housed in a pathogen-free super clean barrier facility environment. All experiments were performed to the National Institutes of Health (NIH) guidelines and approved by the VA San Diego Medical Center IACUC. In addition, for experimental controls, age-matched BALB/c wild type (WT) and NSG mice were also purchased from Jackson.

### Otitis media induced in mice

MEs were infected with nontypeable *Haemophilus influenzae* (NTHi) strain 3655 (nontypeable, biotype II, originally isolated from the ME of a child with OM in St Louis, MO, United States). We have previously used this strain to study OM in various WT and genetically modified mice (Kurabi et al., 2014; Deniffel et al., 2017). The inoculate was prepared and the surgeries performed as previously described (Hernandez et al., 2008). In brief, to induce a ME infection, the bullae were bilaterally exposed *via* a ventral approach through soft tissue dissection and approximately 5 μl of NTHi inoculum was injected with a 30-gauge needle syringe through the hole drilled with a 25-gauge needle. After the injection of NTHi inoculum at dose of 10^4^ Colony forming units (CFUs) per mL, the tympanic membranes were visually confirmed to be intact. The incision was then stapled and the mice were given normal saline and analgesics subcutaneously while recovering on the heated mat. The ME specimens for the 0 h time point were from uninoculated ears. At each time point, blood, bone marrow, and ear tissue were collected for evaluations. In addition, the MEs were cultured for the presence of viable NTHi and evaluated histologically for mucosal hyperplasia and infiltration by leukocytes. Six MEs were evaluated for each mouse strain and time point.

### Morphometry

MEs from control and infected mice were fixed in buffered paraformaldehyde (PFA), decalcified in 10% EDTA for 1 week, and paraffin embedded. Following, the blocks were sectioned at 10 µm and stained with H&E. Mucosal thickness was measured at several standardized locations in the ME, and averaged to obtain a measure of mucosal hyperplasia. Presence of cellular infiltrate in the ME space was recorded.

### Immunohistochemistry

Deparaffinized and hydrated 10 μm ME sections were heated in citrate solution (pH 6.0) for antigen retrieval. After quenching endogenous peroxidase, sections were incubated with anti-hCD45 antibody (DAKO) for 24 h. Immunoreaction was detected by horseradish peroxidase-labeled ImmPRESS anti-mouse/rabbit secondary antibody (Vector Laboratories) developed with 3,3′-diaminobenzidine (DAB) chromogen (Vector Laboratories) and counterstained with hematoxylin (Vector Laboratories). Immunostained slides were imaged with an FSX100 microscope and exposure-matched pictures from humanized vs. NSG and BALB/c control mice compared.

### Bacterial clearance

Bacterial presence in the ME was evaluated by obtaining a sample from the ME lumen using a sterile 1 μl loop. This culture was streaked sequentially onto four quadrants of a chocolate agar plate. The plates were then incubated overnight (16–18 h) at 37°C. Plates were scored based on the observation of any NTHi growth as positive or negative. A colonization score (CS) was used to assess the CFUs/mL present: 0 indicated no CFUs on the plate and 4 indicated CFUs in all four quadrants on the plate. In addition, all colonies on the plate were totaled to obtain CFUs/mL.

### Flow cytometry

Peripheral blood, bone marrow, and infiltrating cells flushed from ME cavity were collected in 1% Heparin (USP) and added to 2 ml 1x ACK-FACS lysis buffer (Invitrogen). Cells were collected by a short spin at 500 *g* followed by a live/dead staining with 7AAD (Invitrogen, CA). The cell suspensions were stained with fluorochrome-conjugated antibodies against human CD45 (BD Biosciences), mouse CD45 (BD Biosciences) and/or stained for various cell surface markers. Cells were assessed with a flow cytometer, BD FACSCanto™ RUO Special Order System with 405, 488, & 640 Lasers. The software is BD FACSDiva™ Software version 6.1.3.

### RNA extraction and RT2 real-time profiler PCR array

Total RNA was extracted from ME tissue and cellular infiltrate using TRIsol (Invitrogen, Carlsband, CA) followed by the RNeasy kit (Qiagen, MD, United States) for purification. The RNA was reverse-transcribed using the RT^2^ First Strand Kit (Qiagen) according to manufacturer’s guidelines. A total of 0.5 μg of RNA was used for each reaction; quantified using Nanodrop (ThermoFisher Scientific, Waltham, MA). Synthesized cDNA was mixed with the RT2 SYBR Green Mastermix and aliquoted into the wells of the RT2 profiler PCR Array (Qiagen, Hilden, Germany) for human innate and adaptive immune responses (PAHS-052ZA). PCR was performed as indicted by manufacturer to evaluate the expression of specific human genes. The plates were run in a StepOnePlus (Applied Biosystems, Waltham, MA) thermocycler and the cycling conditions were set to 95°C for 10 min, followed by 95°C for 15 s, and 60°C for 1 min, for 40 cycles. The threshold cycle (C_T_) value for each sample was then extracted using the automated cycler software and amplification plots. The exported C_T_ values were analyzed using Qiagen RT2 Profiler data analysis software. The “Data QC” section was reviewed to evaluate each group’s PCR reproducibility, reverse transcription efficiency and presence of genomic DNA contamination. Per assay plate, a set of five housekeeping genes was included for data normalization. Statistical analyses were done according to the manufacturer’s instruction and data analysis portal. Gene expression more than twofold and *p* < 0.05 were considered significant.

## Results

### Confirmation of engraftment

Humanized mice have been previously utilized to examine various infectious disease models but they have not yet been applied to OM. We initiated our investigation by confirming levels of engraftment of the huNSG mice with human CD45^+^ cells. Peripheral blood was subjected to flow cytometric analysis using an antibody to human CD45 to detect circulating levels of human CD45^+^ cells (hCD45^+^). Percent human cell engraftment at the time of OM initiation ranged from 19%–67% of total viable circulating cells (Figures 1A,B).

### Histological and bacteriological analysis of uninfected humanized mouse middle ears

Various opportunistic bacteria in mice can spontaneously cause OM. Since NSG mice are severely immune deficient, it was important to investigate the ME to exclude predisposition to OM or any abnormalities in the ear structure. Initial otoscopic examination of 12 week-old humanized NSG mice showed no signs of ME infection; i.e. no reddened or bulging tympanic membranes or fluid in the ME. Similar results were observed in the age-matched BALB/c and C57/BL6 WT experimental controls. In addition, ME sections from the different mouse strains were stained and evaluated for bacterial contamination by Gram staining. No spontaneous infections were observed in the huNSG mice (Figure 1C). Finally, PCR was performed for ribosomal RNA of NTHi, which proved to be negative in all untreated mice strains (results not shown).

### Pathology of the middle ear in a humanized NSG mouse model of otitis media

**FIGURE 2:**
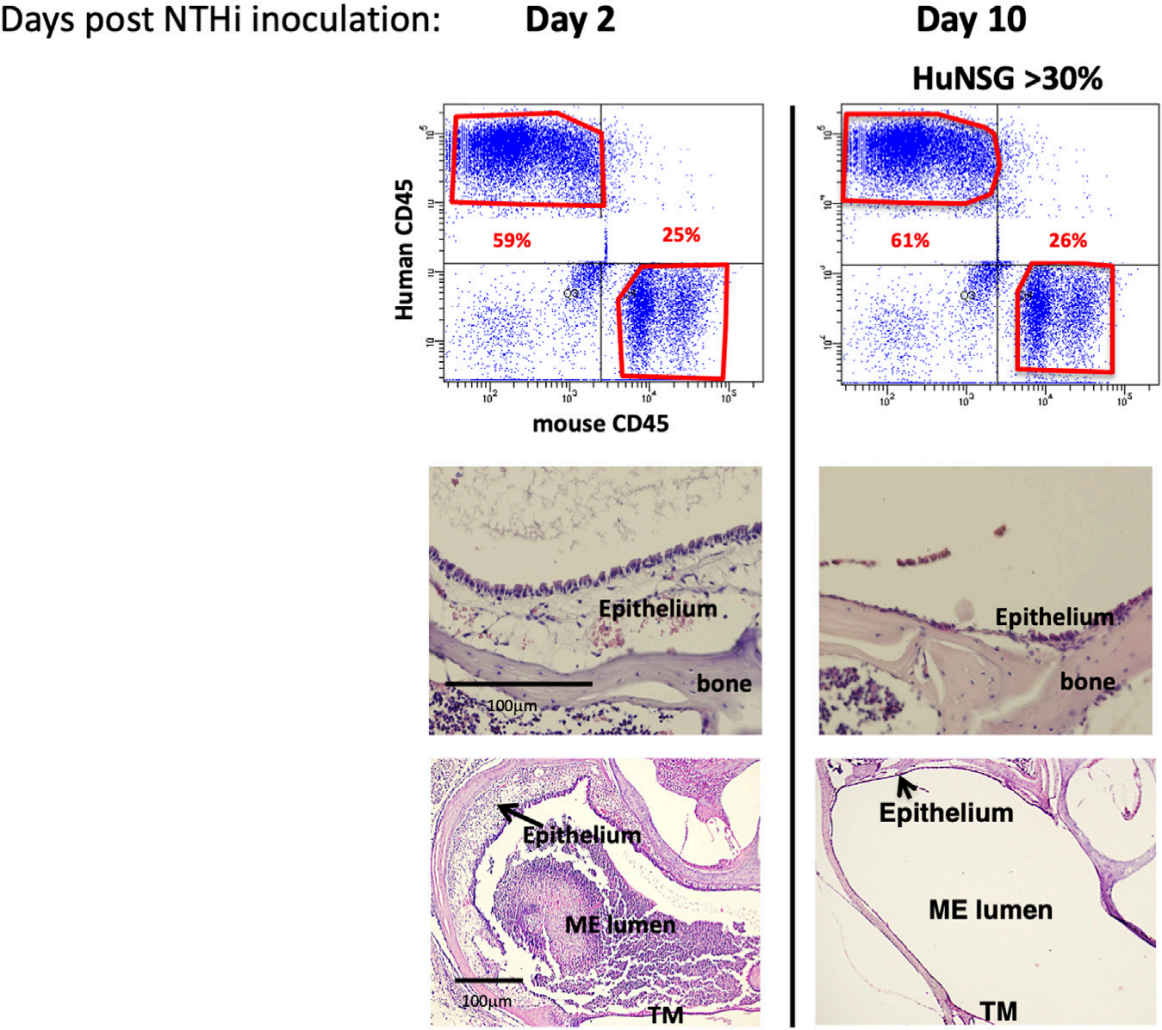
NTHi produced a functional ME immune/inflammatory response in humanized NSG mice. Examination of huNSG mice ME response to NTHi challenge at 2 and 10 days post infection (*n* = 4 mice). Top: comparison of mCD45^+^ vs. hCD45^+^ cells in the blood by flow cytometry analysis confirming >30% engraftment. Bottom: Histopathological changes in ME showing significant mucosal thickening and ME space leukocytic infiltration at 2 days after ME infection, and recovery prior to day 10.

To assess whether NTHi elicits a functional ME immune/inflammatory response in huNSG mice, we used standard methodology (Hernandez et al., 2008) to inoculate the MEs of C57/BL6, BALB/c and huNSG mice with NTHi and ME was characterized for immune/inflammatory responses. We assessed ME histopathology at 48 h (the peak of normal mouse OM) and at 10 days (to evaluate OM resolution and bacterial clearance). The histopathology observed in BALB/c (WT) mouse MEs is shown in Figure 2. At 48 h, the ME mucosa exhibited hyperplasia accompanied by a robust leukocytic infiltrate, as we have reported previously in C57/BL6 (Kurabi et al., 2014; Deniffel et al., 2017). Similarly by 10 days, the WT MEs had recovered to their normal appearance.

**FIGURE 3:**
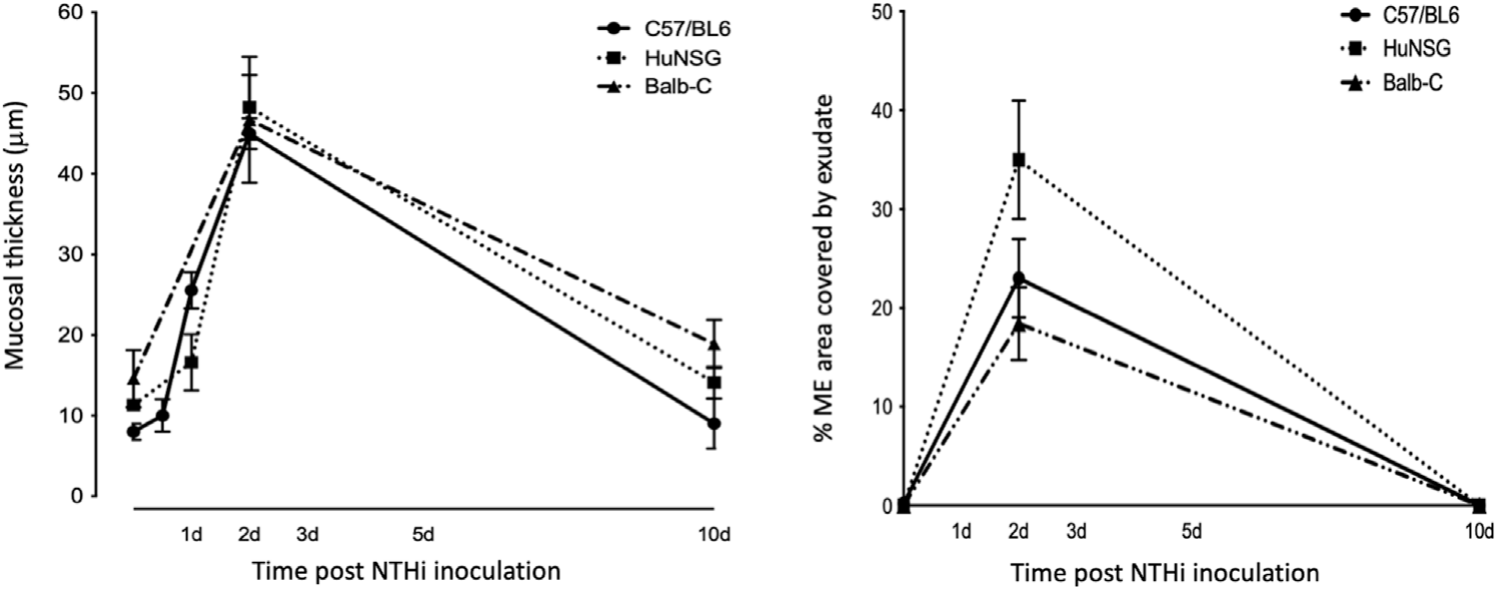
Quantitative comparison of mucosal hyperplasia and leukocyte infiltration in to the ME cavity post NTHi challenge. Both humanized mice and WT mice showed comparable mucosal hyperplasia and infiltration of the ME by leukocytes (% occupied area of ME by cells) 48 h after NTHi inoculation. All Balb/c, C57/BL6 and huNSG resolved OM by 10 days.

**FIGURE 4:**
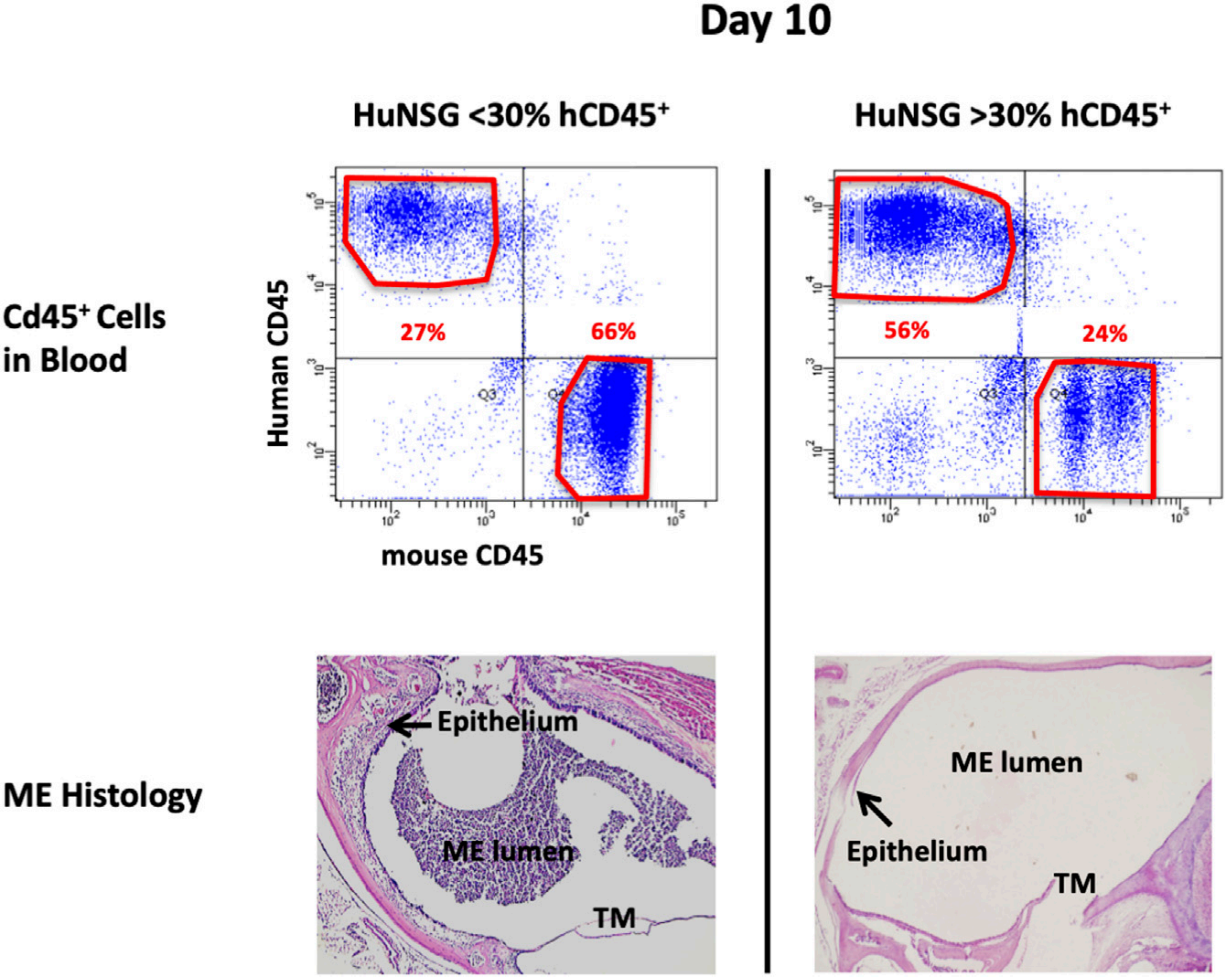
Degree of engraftment levels with human CD45^+^ cell impacts effective and appropriate bacterial clearance. Representative histological images of the middle ear of HuNSG mice that had less than 30% human CD45^+^ leukocytes (in blood) appeared incompetent to clear OM by 10 days. Humanized mice that had greater than 30% human CD45^+^ cells in blood however, appeared to have resolved OM by day 10.

At 48 h after NTHi inoculation, mucosal hyperplasia and inflammatory cell infiltration in all huNSG mice was equivalent to that seen in WT mice. Moreover, by day 10, in huNSG mice showing greater than 30% engraftment, the ME was cleared of immune cells and the mucosa had recovered to its normal appearance (Figure 3). However, in four humanized animals that were found to show <30% hCD45^+^ cell engraftment, the MEs at 10 days exhibited substantial mucosal hyperplasia and leukocyte infiltration, indicating persistent OM (Figure 4).

### Humanized NSG mice support the recruitment of human leukocytes into the middle ear

**FIGURE 5:**
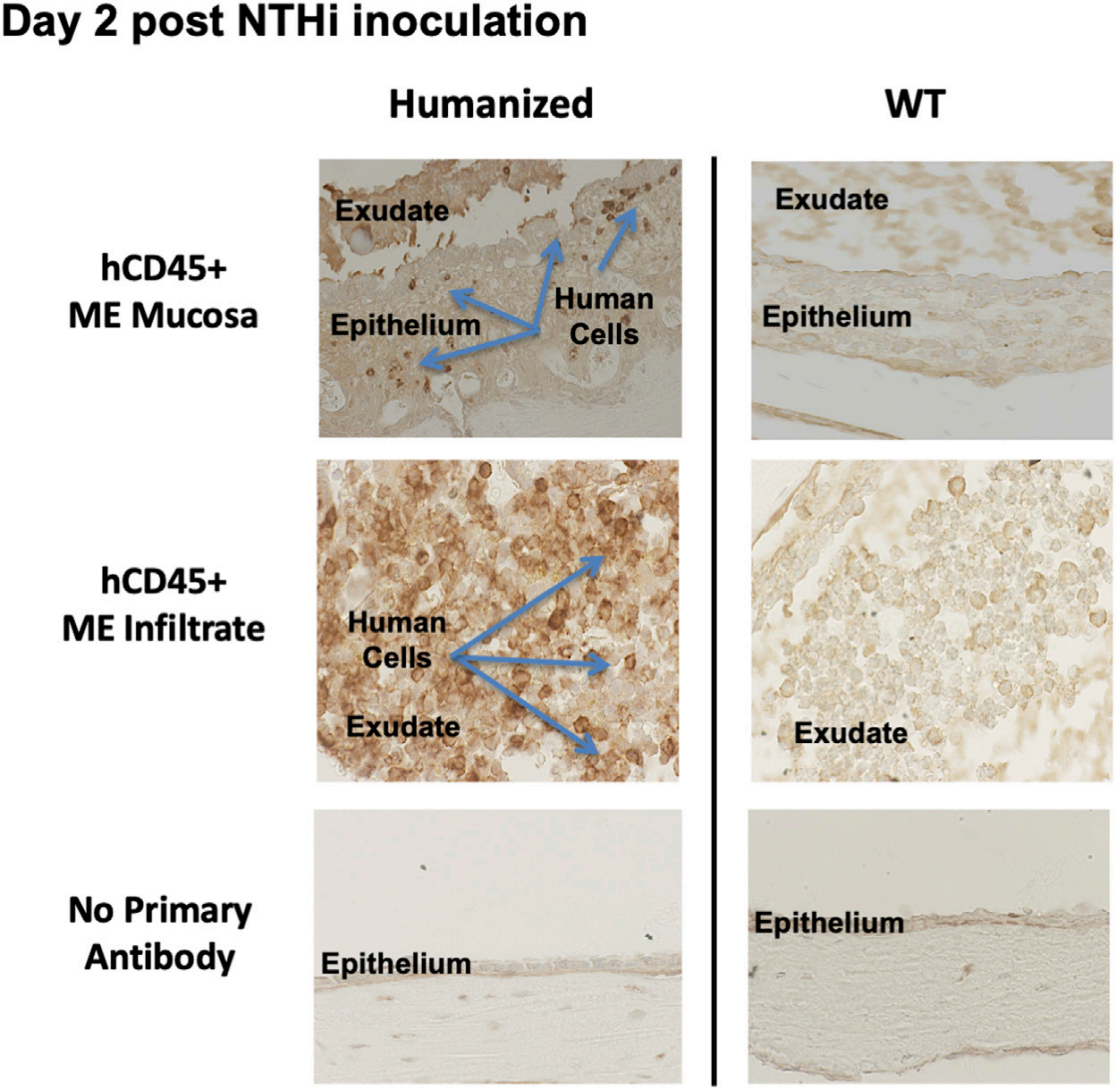
Human CD45^+^ cells behave like mouse CD45^+^ cells in the infiltration of ME space and support appropriate inflammatory response. Immunohistological staining by human leucocyte Common Antigen (hCD45^+^, DAKO) of ME sections at day 2 post NTHi infection showing that human CD45^+^ immune cells were detected in mucosa and ME effusions of mice with humanized immune systems but not WT. Infiltration of hCD45 positive cells into the ME mucosa and lumen strongly suggests that the human immune cells are playing a role in both ME inflammation and NTHi bacterial clearance.

At 48 h after inoculation, human immune cells were found to be present in the ME cavity of huNSG, as indicated by hCD45 immunohistochemistry (Figure 5). Infiltration of hCD45^+^ cells into the ME mucosa and lumen strongly suggests that the human immune cells are playing a role in both ME inflammation and NTHi bacterial clearance, since NSG mice lack immune responses to bacterial and fungal pathogens (Foreman et al., 2011).

### Middle ear bacterial clearance during otitis media in WT and huNSG mice

At 48 h after NTHi inoculation, all WT and huNSG MEs were culture-positive. The high numbers of bacteria recovered at this timepoint is indicative of bacterial replication and persistence, namely infection of the ME. At 10 days, all MEs of WT and huNSG mice with >30% engraftment were culture-negative, indicating recovery from bacterial OM. In contrast, MEs of mice showing <30% engraftment were all culture positive at 10 days (Table 1).

**TABLE 1:** Bacterial clearance of middle ears for different mice strains through the experimental time course of OM.

Time after NTHi inoculation	BALB/c mice		Humanized mice >30% hCD45^+^ cells		Humanized mice <30% hCD45^+^ cell	
	Mean bacterial CS	Mean CFUs	Mean bacterial CS	Mean CFUs	Mean bacterial CS	Mean CFUs
Day 2	3.5	8 × 10^3^	4	1 × 10^4^	4	1 × 10^4^
Day 10	0	0	0	0	4	1 × 10^5^

Bacterial colonization of chocolate-agar plates was evaluated using a semi-quantitative method to generate colonization score (CS) as follows: 0 indicated no colony-forming unit (CFU) on the plate; 1 indicated CFUs in one quadrant; 2 indicated CFUs in two quadrants; 3 indicated CFUs in three quadrants; and 4 indicated CFUs in all four quadrants on the plate. At least 6 ears were cultured for each time points of WT and humanized mice.

### Detection of human genes in the middle ears of huNSG mice

To study the expression of human-specific genes in huNSG mouse ears, ME tissue and infiltrating cells were recovered from MEs of huNSG mice and/or control NSG mice (unengrafted) and processed for RT-PCR. We utilized the Qiagen RT2 profiler PCR array for human innate and adaptive immune responses. This array has a focused panel of 84 human genes related to host responses during bacterial infections. Some of those genes are human-specific, having no mouse orthologs. This includes CCL2 (MCP-1), HLA-A, HLA-E, IFNA1, and CXCL8 (IL8). For the remainder, the array detects a human-specific sequences in the mRNA. To ensure that no probes were cross-reacting with NSG mouse genes, uninfected (0 h) and 48 h infected unegrafted NSG samples were also run and included in the analysis. We assessed only those genes in which there was no detectable expression in the MEs of unengrafted NSG mice. There was a number of human genes that had some basal gene expression in the huNSG mice ME but were not detected (C_T_ value greater than 35 cycles) in the unegrafted NSG mice at 0 h; i.e. prior to OM induction.

**FIGURE 6:**
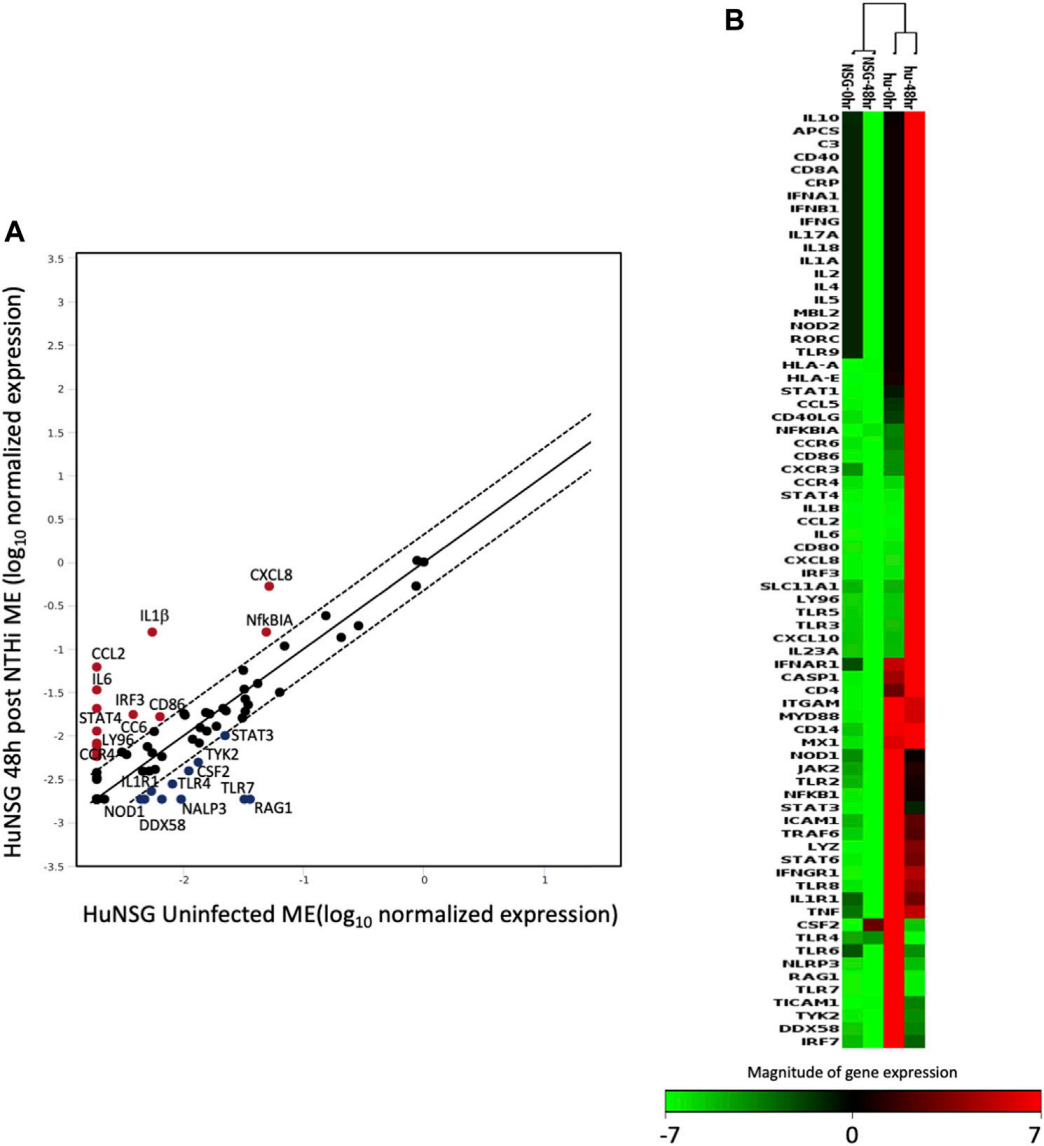
The expression change of 84 human genes related to inflammatory pathways in humanized mice after NTHi inoculation was evaluated by real-time PCR based array analysis (Qiagen). **(A)** Scatter Plot representing the expression level of each human gene in huNSG ME at 48 h post NTHi infection vs. uninfected huNSG (0 h). Genes above and below the red lines are regulated more than two-fold (up or down) in 48 h infected vs. 0 h samples (*n* = 3). **(B)** Non-supervised hierarchical clustering of the entire dataset (huNSG at 0 h and 48 h plus unengrafted NSG controls at 0 h and 48 h) providing an overview of gene expression patters as a continuous heat-map based on magnitude of gene expression. Red represents high expression, while green represents low expression and black is no change. NSG control data (first two columns) have low to no expression (green/black color) indicating that the array probes did not cross-react with mouse RNA. The rows represent genes evaluated by the Qiagen RT2 array.

The human inflammatory responses could be selectively assessed after induction on OM by comparing the RNA gene expression patterns from ME tissue of huNSG collected at 0 h to those that had been inoculated with NTHi at 48 h. Figure 6 shows a scatter plot analysis (A) presenting the genes that were differentially expressed among huNSG mice at the different time points, 0 h vs. 48 h. Gene’s in red represent up-regulated genes while blue represents down-regulated genes (2-fold cutoff). In Figures 6B, a clustergram heat-map comparing the gene profiles of huNSG vs. unengrafted NSG at the 0h and 48 h NTHi inoculation time points reveals that many human genes were specifically expressed in the huNSG mice and that the human immune cells in this mouse model play a role in the immune responses. There were very little changes in the gene expression levels in the unengrafted NSG mice at 0 h vs. 48 h.

### Comparison of huNSG mice gene responses to human otitis media

We compared RT^2^ PCR results from our huNSG mice to transcriptome data from human pediatric OM caused by NTHi (Liu et al., 2013). We found that many genes related to immunity and host defense were regulated in the middle ear mucosa of huNSG mice at 48 h post NTHi infection (Table 2). We next compared these data to published array data profiling the gene expression of PBMCs isolated from children with NTHi AOM infections relative to the pre-infection healthy state in the same children (Liu et al., 2013). TLR7 and DDX58 were down-regulated in both the huNSG and human PBMCs during OM but not in the regular WT mice (Hernandez et al., 2015). Of Note, the human transcript data are not from the ME, and are not timed the same relative to infection.

**TABLE 2:** Comparison of human genes with a >3 Fold difference in the middle ear of huNSG mice at 48 h post NTHi infection, with those same gene expression from WT mice (Hernandez et al., 2015) at same time, and human PBMCs isolated from pediatric OM patients (Liu et al., 2013). (FC, fold change; NA, data not available, positive values indicate up-regulation and negative down-regulation).

Gene	HuNSG ME FC	Human PBMCs FC	WT mouse ME FC
CCL2	65.33	−11.11	54.8
TLR7	−64.59	−3.13	3.13
RAG1	−59.10	2.26	NA
IL6	39.39	−3.70	21.47
STAT4	26.90	NA	3.37
IL1B	24.31	−6.25	17.04
NLRP3	−18.13	NA	3.43
DDX58	−13.26	−2.08	1.00
CD80	10.30	NA	7.06
NOD1	−8.71	NA	1.19
TLR5	8.44	NA	NA
CXCL8	7.86	NA	NA
IL23A	7.86	NA	NA
IL1R1	−7.75	4.65	NA
CXCL10	7.58	−100	6.03
TBX21	6.55	NA	NA
CCR4	6.40	NA	NA
IL13	−5.03	NA	NA
IL10	4.33	NA	3.96
IRF3	4.18	NA	NA
IFNG	4.10	NA	NA
CCR5	−3.88	NA	7.29
CCR8	−3.70	NA	NA
TLR1	−3.53	NA	2.72
CSF2	−3.38	NA	26.04
TYK2	−3.01	NA	NA
MPO	−3.01	NA	NA

## Discussion

Mouse models have provided the research community with indispensable tools for studying human diseases, including OM. With over 17,000 orthologous genes between the mouse and human genomes, much has been discovered about the basic immune, inflammatory and genetic mechanisms of OM using murine models. However, there remain a large number of human genes that lack murine homologs or are differentially regulated in humans. In particular, many variations between human and mouse immune responses have been documented [e.g., (Mestas and Hughes, 2004; Gibbons and Spencer, 2011; Zschaler et al., 2014)]. Since many human responses are unique, it is important to closely link animal studies to human immunity and inflammation, and to assess human-specific responses whenever possible.

That said, transcript profiling and gene knockout mice have allowed the identification of many genes and immune pathways involved in OM pathogenesis and recovery (MacArthur et al., 2013; Kurabi et al., 2016; Kyd et al., 2017). This and other evidence strongly suggests that innate immunity is a key response in OM. Normal resolution of acute OM occurs within a few days, with insufficient time for the development of robust adaptive host immunity. Moreover, defects in innate immune genes have been linked to OM persistence in both mice (Kurabi et al., 2016; Bhutta et al., 2017) and humans (Casselbrant and Mandel, 2001; Allen et al., 2014; Verhoeven and Pichichero, 2014). These studies have shown that initiation of the innate immune response against invading microbes is dependent on the integrity of several host pattern recognition receptors and downstream signaling pathways. These signaling cascades mediate the generation and activation of cytokines and chemokines that facilitate a series of immune responses leading to the resolution of infection (Kyd et al., 2017).

We believe that these prior murine data provide an excellent backdrop against which to evaluate the responses of human immune cells in a mouse model. A humanized mouse model allows us to compare and contrast human and murine immune responses in OM, validating the data of murine studies when possible, and identifying unique characteristics of human immune responses when they vary from those of the mouse. Identification of such differences will accelerate the translation of therapies from animals to humans, with improved efficacy and selectivity, since it is clear that many disease treatments developed in mice do not translate well to humans (Bracken, 2009). A successfully established humanized mouse model of OM would provide a foundation for evaluating the responses of immune cells from humans with different OM phenotypes. Such studies would provide valuable information about immune differences that may underlie OM proneness in human patients.

The findings of this study validate humanized mice as an *in vivo* animal model with which to study human immune responses to OM. We have demonstrated that humanized mice demonstrate normal ME inflammatory responses, leukocyte infiltration, tissue hyperplasia, bacterial clearance, and OM recovery evoked by inoculation of the ME with NTHi experimentally; although it is important to note that mice must have a sufficient level of circulating hCD45^+^ cells to mount an effective defense of the ME. In addition to these phenotypic and microbiological studies, RT-PCR identified and profiled human-specific genes that were expressed in the MEs of mice engrafted with HSPCs.

Prior studies have evaluated the immune cells present in the ME fluid (MEF) of OM-prone *versus* OM-resistant children, showing significant recruitment of CD4^+^ T-helper cells in addition to the presence of a number of pro-inflammatory mediators that aid in the immune responses (Verhoeven and Pichichero, 2014; Kaur et al., 2015). The transcriptome profile of peripheral blood immune cells in these children has also been evaluated, and differences related to OM proneness identified (Liu et al., 2012; Liu et al., 2013). These prior data suggest that engraftment of mice with human immune stem cells from OM-prone *versus* OM-resistant children would yield information on human ME immune processes that are critical for OM resistance and resolution, across the entire duration of the infection. They could also serve as models with which to test the ability of treatments to boost or supplement immunity and improve OM resistance.

Humanized mice can be used to address critical questions that cannot readily be accomplished in patients. First, this model allows the characterization of human immune responses in the early stages of OM, which are typically not accessible in patients who present only when symptomatic. The humanized mouse can thus be used to help identify the disease’s initiating factors. Second, infection of the ME can be carefully controlled in a mouse model, so that human immune responses against defined pathogen populations and titers can easily be evaluated. Third, the humanized mouse permits ready access to immune cells from the ME as well as from peripheral blood, so that interactions between the cells and the microenvironment of the ME can be assessed throughout the course of OM.

Some limitation to using humanized mice however need to be considered and the engraftment technology still needs further development. These engrafted mice are heterogenous in their degree of humanization and cannot be bred to reproduce. The mice are used at 12–16 weeks of age and the stability of the human engraftment with age plays a role in the immune responses. The cost for producing these mice and purchasing them is still high (∼$400 to $1,200 per mouse) for a conventional laboratory model. Finally, induction of OM by direct inoculation of a single bacterium into the ME is rather not similar to clinical human disease where the pathogenesis involved nasopharyngeal colonization followed by ascent to the ME and polymicrobial infection (Bakaletz, 2010; Massa et al., 2021). It has been shown that the host responses vary by pathogen species [e.g., (Melhus and Ryan, 2000; Liu et al., 2013)]. We note that our study only probed host responses against NTHi. The effects of infection with other bacterial strains such as *Moraxella catarrhalis* or *S. pneumoniae* were not studied.

## Conclusions

We have developed a humanized mouse model that recapitulates the normal course of acute OM using NTHi, a human pathogen that is among the most common causes of ME infection. NTHi-induced OM in huNSG mice resembles that seen in immunocompetent WT mice. However, the model includes the infiltration of human immune cells into the ME, where they express human-specific genes and mediate recovery from infection. Humanized mice thus provide the opportunity to perform a wide variety of studies on human immunity in OM. They can also be used as a preclinical tool to assess the immune proneness to this common disease.

## Data Availability

The raw data supporting the conclusion of this article will be made available by the authors, without undue reservation.
